# Supplementary figures and images for: The Pleiotropic Effects of YBX1 on HTLV-1 Transcription
Source: Int J Mol Sci. 2023 Aug 23;24(17):13119. doi: 10.3390/ijms241713119 (PMC10487795; doi:10.3390/ijms241713119)

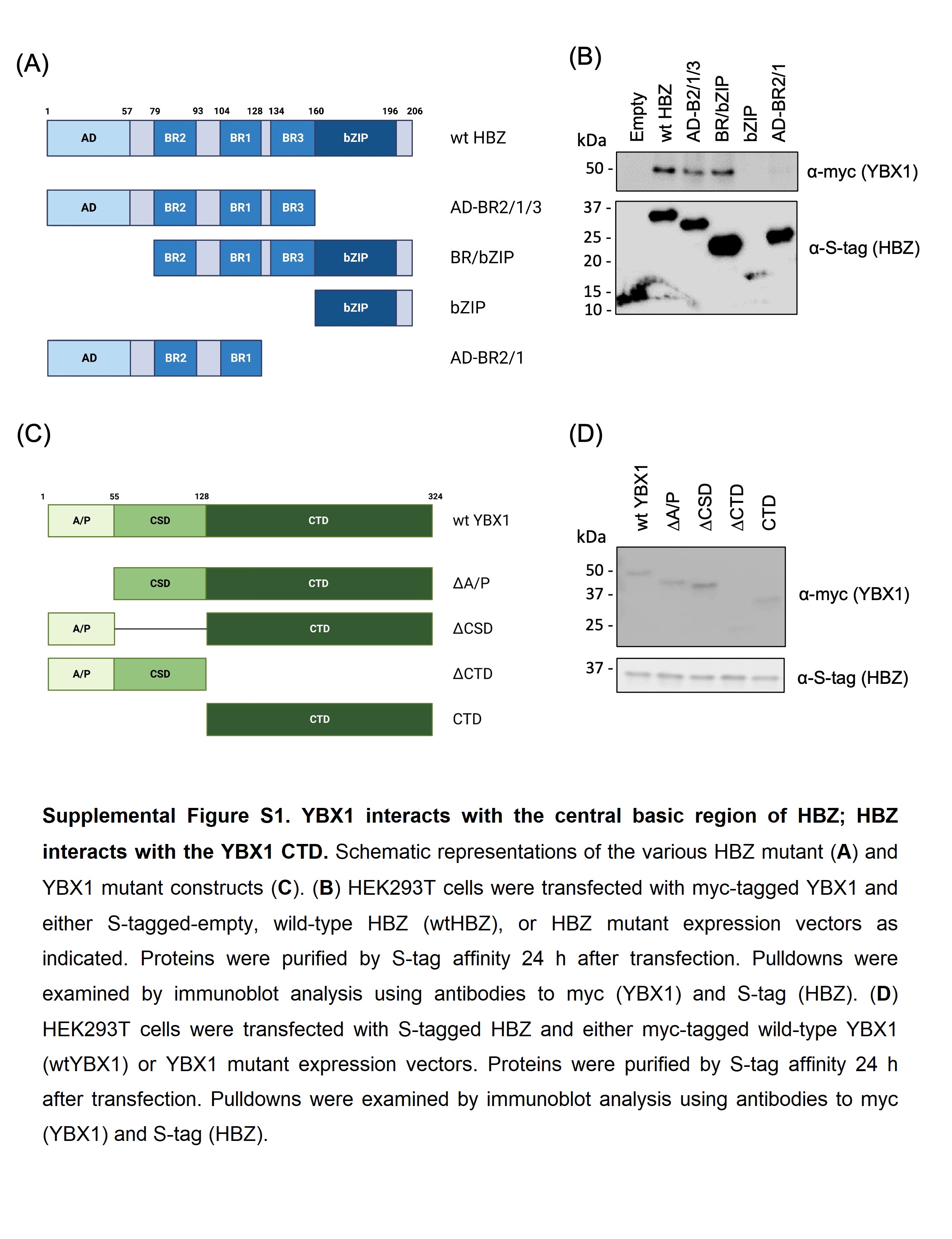

Supplement: Supplementary file 1 [file ijms-24-13119-s001.zip › Supplemental Figure S1.tif]
